# Retrograde inferior vena cava perfusion reduces the risk of acute kidney injury depending on the oxygen extraction ratio. A retrospective cohort study

**DOI:** 10.3389/fcvm.2025.1514247

**Published:** 2025-04-28

**Authors:** Xinyi Liao, Dan Luo, Jing Lin, Zhaoxia Tan, Jiyue Xiong, Lei Du

**Affiliations:** Department of Anesthesiology, West China Hospital, Sichuan University, Chengdu, Sichuan, China

**Keywords:** acute type A aortic dissection, acute kidney injury, retrograde inferior vena cava perfusion, antegrade cerebral perfusion, ERO_2_

## Abstract

**Background:**

Total aortic arch replacement surgery (TARS) for Acute type A aortic dissection is associated with high incidence of postoperative acute kidney injury (AKI), at least partly due to the lower body ischemia during circulatory arrest. This study aimed to evaluate whether retrograde inferior vena cava perfusion (RIVP) reduces the risk of AKI by providing oxygenated blood to the lower body.

**Methods:**

This retrospective study utilized a medical recording system to screen patients who underwent TARS from January 1 to December 31, 2019. Patients were assigned to receive antegrade cerebral perfusion (ACP) only or ACP + RIVP during circulatory arrest. The primary outcome was postoperative AKI. Oxygen delivery, consumption, and extraction ratio during RIVP were also determined.

**Results:**

Of all included 87 patients, postoperative AKI occurred in 35 (40%), of whom 23 (53.5%) were in the ACP, and 12 (27.3%) were in the ACP + RIVP (*P* = 0.013). In regression analysis, ACP + RIVP was associated with lower risk of AKI than ACP alone (adjusted OR 0.229; 95% CI 0.071–0.746). RIVP at a pressure of 22.5 ± 3.8 mmHg delivered 0.98 ± 0.34 ml/min/kg of oxygen to the lower body, and the partial oxygen pressure decreased from 359 ± 57 mmHg in RIVP blood to 64 ± 30 mmHg in returning blood. Oxygen extraction ratio was 44 ± 16%, which correlated negatively with peak postoperative creatinine levels (*r* = −0.58, *P* = 0.01) and creatinine increase (*r* = −0.61, *P* = 0.009). No correlations were found between oxygen delivery and postoperative creatinine or creatinine increase.

**Conclusion:**

RIVP may reduce the risk of postoperative AKI in a manner that depends on the tissue oxygen extraction ratio.

## Introduction

Acute type A aortic dissection (AAAD) is associated with mortality rates of 27%–45% (1–3). To reduce mortality, this condition is often immediately treated by replacing both the ascending aorta and the aortic arch with artificial vascular grafts under cardiopulmonary bypass (CPB) in a procedure called total aortic arch replacement surgery (TARS) (4). TARS requires a period of circulatory arrest during which arteries are reconstructed via anastomosis of the graft and the proximal descending aorta. This arrest period can cause ischemic injury in vital organs. The application of deep hypothermic circulatory and antegrade (ACP) or retrograde cerebral perfusion can mitigate cerebral ischemic injury (5–7). These techniques, however, still expose the lower body to substantial risk of ischemic injury. As a result, acute kidney injury (AKI) occurs in up to 54.5% of TARS patients (4, 8, 9).

Our group proposed a technique combining ACP with retrograde inferior vena cava perfusion (RIVP), which may provide oxygenated blood to the lower body during circulatory arrest in TARS (10, 11). Theoretically, RIVP may avoid ischemic injury of the low body during circulatory arrest, but whether it can reduce risk of AKI has not been explored in detail. Therefore, the present study utilized a medical recording system, tested whether RIVP reduces risk of AKI and its potential mechanism.

## Materials and methods

### Study subjects and ethics

Patients aged ≥18 years who were diagnosed with AAAD and underwent TARS with ACP combined with RIVP during circulatory arrest at West China Hospital between 1 January, 2019, and 31 December, 2019, were eligible for this study. Patients were excluded if they were pregnant or had renal dysfunction requiring dialysis before surgery. This single-center retrospective cohort study was approved by the Biomedical Ethics Committee of West China Hospital (2021–837). Because of the study's retrospective nature, the informed consent requirement was waived.

### Patient and public involvement statement

Patients or the public were not involved in the design, or conduct, or reporting, or dissemination plans of our research.

### Data collection

Data on demographic and clinical characteristics, perioperative details, and blood analysis results were extracted from our hospital's electronic records system. Blood gas analysis values obtained from anesthesia and CPB record sheets. Information on patients undergoing ACP or ACP + RIVP was also available on the CPB record sheets. All preoperative examinations and test results closest to the operation day were included.

### Anesthesia and surgical procedures

The procedures were performed according to our hospital's protocol. Briefly, patients received sevoflurane via inhalation along with intravenous infusions of propofol, sufentanil and cisatracurium. Cannulation for systemic perfusion was performed in the aortic arch, right axillary artery or femoral artery (Supplementary Table S1), according to the vascular dissection and surgeon's preferences. The superior and inferior venae cavae were cannulated for venous drainage. After cross-clamping the ascending aorta, cardiac arrest was achieved by intermittent antegrade infusion of cold blood cardioplegia and maintained using either antegrade or retrograde delivery methods.

Systemic perfusion was stopped and circulatory arrest was initiated when the nasopharyngeal temperature reached 24–26°C and the rectal temperature fell below 28°C. The aortic clamp was removed to open the aorta, and a four-branched artificial graft was employed for TARS. An elephant trunk was also inserted into the descending aorta, if necessary. After anastomosis of the graft with the descending aorta and left subclavian artery, systemic perfusion was performed via the fourth branch, and rewarming began.

The arch was reconstructed by anastomosis of the left common carotid artery and the innominate artery with the other two side branches of the hybrid prosthesis. Patients were progressively weaned off CPB.

In accordance with standard protocols at our hospital, red blood cells were transfused when hemoglobin concentration was <7 g/dl during CPB, <8 g/dl during surgery or <9.5 g/dl in the ICU.

### ACP and RIVP treatment

For ACP, supra-aortic vessels were gently clamped during circulatory arrest, and ACP was achieved by axillary cannulation or direct innominate artery cannulation with a cannula (10–14 Fr) at 5–12 ml/kg/min under pump pressure of 50–80 mmHg. The systemic perfusion was initiated from the graft after anastomosis of the descending aorta and the graft (5, 12, 13).

The RIVP procedure involved securing the inferior vena cava using a perivascular band near the cannulation site, combined with distal occlusion of the venous drainage catheter. This configuration enabled retrograde perfusion through another pump delivering oxygenated blood into the venous system. Technical specifications for this protocol are detailed in our team's prior publication (10). The flow of RIVP was regulated to achieve a target venous pressure of 20–25 mmHg. This range of pressure was selected assuming that pressures below 20 mmHg may result in insufficient organ perfusion, while pressures exceeding 25 mmHg could lead to ascites (14). A pressure monitoring tube was connected to the pipeline distal to the roller pump, which was infused into the inferior vena cava, to measure RIVP pressure. RIVP was discontinued after anastomosis, and systemic perfusion was initiated from the graft as described for the ACP group.

### Blood gases data and lactate

In the beginning of 2019, we detected the blood samples simultaneously from the arterial line and the descending aorta under the assistance of a surgeon in some patients with RIVP, then analyzed immediately on a blood gas analyzer (Cobas b 123; Roche, Basel, Switzerland), which allowed us to determine the oxygen delivery (DO_2_) and, oxygen consumption (VO_2_) of RIVP. The following data were then collected: oxygen partial pressure (PO_2_), oxygen saturation (SO_2_), and hemoglobin levels.

Oxygen content of the arterial line (CaO_2_) and of venous blood from the descending aorta (CvO_2_) was calculated using the equations (15):

(1) $$C_{a}O_{2}=(P_{a}O_{2}\times 0.00315)+(Hb\times 1.34\times S_{a}O_{2})\;(\mathrm{ml}/L)$$

(2) $$C_{v}O_{2}=(P_{v}O_{2}\times 0.00315)+(Hb\times 1.34\times S_{v}O_{2})\;(\mathrm{ml}/L)$$

where $P_{a}O_{2}$: arterial partial pressure of oxygen (mmHg); $P_{v}O_{2}$: venous partial pressure of oxygen (mmHg); Hb: hemoglobin concentration (g/dl); $S_{a}O_{2},S_{v}O_{2}$: arterial and venous oxygen saturation (%)

DO_2_, VO_2_ and oxygen extraction ratio (ERO_2_) were calculated using the equations (15):

(3) $$DO_{2}=C_{a}O_{2}\times Q/W\;(\mathrm{ml}/\min/\mathrm{kg})$$

(4) $$VO_{2}=(C_{a}O_{2}-C_{v}O_{2})\times Q/W\;(\mathrm{ml}/\min/\mathrm{kg})$$

(5) $$ERO_{2}=VO_{2}/DO_{2}\times 100\;(\text{\%})$$

where Q is RIVP blood flow rate (L/min), and *W* is the patient's weight (kg).

Plasma lactate levels before CPB (baseline), before circulatory arrest, 5 min after lower body perfusion was restored, after rewarming to 32°C and at the end of CPB, were also collected. The increase in lactate was calculated as the lactate level at the end of CPB minus lactate level before CPB.

### Outcomes

The primary outcome was the occurrence of postoperative AKI before hospital discharge. AKI, which was defined using the Acute Kidney Injury Network classification (16). AKI stage 1 was defined as an increase in serum creatinine ≥27 mol/L or an increase from baseline ≥150%. Stage 2 was defined as an increase in serum creatinine value ≥200% from baseline. Stage 3 was defined as an increase in serum creatinine ≥300% from baseline, an absolute serum creatinine level ≥354 mol/L, or requirement of dialysis.

Serum creatinine and urea nitrogen levels were collected before surgery (baseline), daily in the intensive care unit, and in the ward after surgery. Peak creatinine was defined as the postoperative maximum, and its increase was defined as the difference between the peak and the baseline.

Secondary outcomes were indices of oxygen metabolism during RIVP, including PO_2_ in the returned blood, DO_2_ of RIVP, as well as VO_2_ and ERO_2_ in the lower body.

Other outcomes were occurrence of the following composite adverse events after surgery: all-cause death, defined as death for any reason within 30 days after surgery or to hospital discharge, if the hospital time over 30 days; stroke, defined as an acute episode of focal or global neurologic dysfunction caused by brain injury as a result of hemorrhage or infarction in which the neurologic dysfunction lasts for >24 h (17); prolonged ventilation >24 h (17).

### Statistical analysis

Continuous variables were reported as mean and standard deviation or median and 25th and 75th quartiles, and categorical variables as frequency and percentage. In the case of continuous variables, differences between groups were assessed for significance using the unpaired *t* test if the data showed a normal distribution, or the rank sum test if data were skewed. In the case of categorical variables, differences were assessed using the chi-squared test or Fisher's exact test when the theoretical frequency was less than 5. Pearson or Spearman correlation analysis was performed to examine the association between oxygen metabolism and plasma creatinine. Univariate and multivariate logistic regression was applied to calculate the odds ratios (ORs) and 95% confidence intervals (CIs) of ACP + RIVP relative to ACP patients. Data were analyzed using SPSS 22.0 (IBM Corp., Armonk, NY, USA) and GraphPad Prism version 9 (GraphPad Inc., San Diego, CA, USA). All *P*-values were two-sided, and *P* < 0.05 was considered significant.

## Results

### Patient characteristics

**Figure 1:**
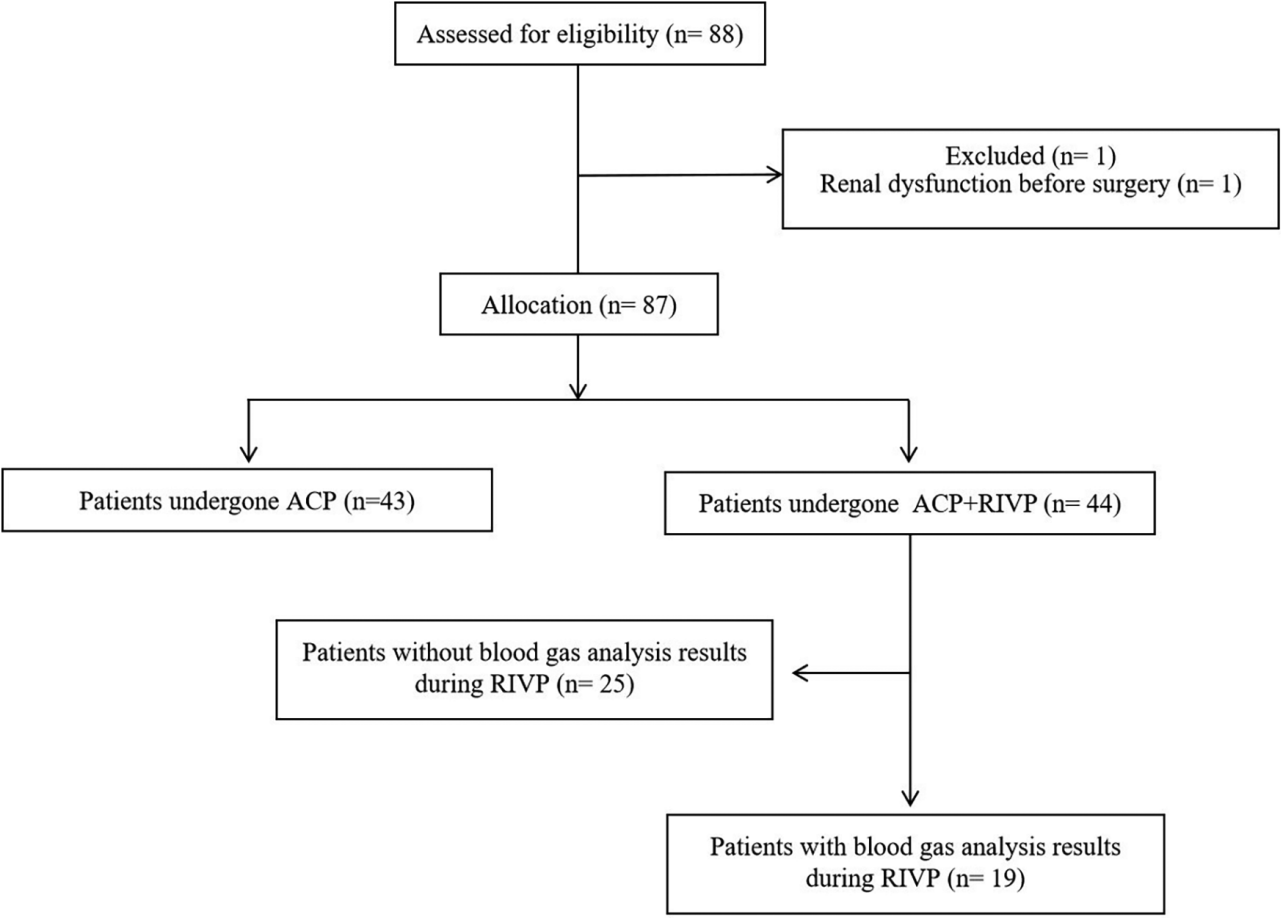
Flow diagram showing patient selection. ACP, antegrade cerebral perfusion; RIVP, retrograde inferior vena caval perfusion.

From January 1, 2019 to December 31, 2019, 88 patients with AAAD undergoing TARS were identified from the medical record system in the Division of Thoracic and Cardiovascular Surgery at West China Hospital. Of them, 1 patient was excluded because of preoperative dialysis. Of the remaining 87 patients, 43 patients with only ACP, and 44 patients with combination ACP and RIVP. Blood gas analyses of blood from inferior vena cava and the descending aorta were available in 19 of the 44 patients (43%), and they were included into calculating oxygen supply and consumption in the lower body during RIVP (Figure 1).

Of all 87 patients, the mean age was 45 years old, 75 (86%) were male, and 53 (61%) had hypertension. The demographic characteristics, including preoperative serum creatinine levels and renal artery involvement in dissection, were comparable between the two groups (Supplementary Table S2).

The surgical procedures, times of aortic cross-clamping and circulatory arrest were also comparable between the groups. However, CPB duration was shorter in the ACP + RIVP group (250 ± 53 vs. 276 ± 58 min, *P* = 0.031) (Supplementary Table S3).

During circulatory arrest, the lowest nasopharyngeal and rectal temperatures were higher in the ACP + RIVP group than in the ACP group (mean difference: 1.7°C, *P* < 0.001). ACP flow was 6.4 ± 2.1 ml/min/kg in the ACP group and 7.2 ± 2.2 ml/min/kg in the ACP + RIVP group at a pump pressure of 50–80 mmHg. In ACP + RIVP patients, RIVP blood flowed during circulatory arrest at 9.6 ± 3.2 ml/min/kg (range 2.54–16.34 ml/min/kg) at a pressure of 22.5 ± 3.8 mmHg (range 15–28 mmHg). Patients in the ACP group received more red blood cells than ACP + RIVP patients (*P* = 0.01, Supplementary Table S3).

DO_2_ did not differ between groups pre-arrest but was higher in the ACP + RIVP group post-arrest (6.2 ± 1.0 vs. 5.4 ± 1.3 ml/kg/min, *P* = 0.001) (Supplementary Table S3).

We obtained blood gas analyses results of the descending aorta during circulatory arrest from 19 of 44 (43%) RIVP patients for determination of oxygen metabolism. The demographic characteristics and perioperative data of these 19 patients were similar to those of the patients from whom without blood gas analyses results.

### Acute kidney injury and other outcomes

Baseline renal function was comparable between groups (Supplementary Table S2). After surgery, AKI occurred in 35 out of 87 (40%) patients before hospital discharge, and it was classified as stage 1 injury in 19 patients, stage 2 in 11 patients, and stage 3 in 5 patients. The incidence of AKI was higher in the ACP group (23/43, 53.5%) than in ACP + RIVP patients (12/44, 27.3%; *P* = 0.014). In the logistic regression analysis, ACP + RIVP was associated with lower risk of AKI than ACP only (crude OR 0.326, 95% CI 0.130–0.785, *P* = 0.014). This risk reduction remained significant (adjusted OR 0.196, 95% CI 0.038–0.843, *P* = 0.036) after adjusting by sex, age, body mass index, hypertension, diabetes mellitus, New York Heart Association class, baseline creatinine level, renal arteries involved in dissection, bypass duration, red blood cell transfusion, lowest nasopharyngeal and rectal temperature (7, 18–20) (Table 1).

**Table 1:** Postoperative acute kidney injury in 87 patients stratified by perfusion strategy during circulatory arrest.

Acute kidney injury, *n* (%)	ACP	ACP + RIVP	Crude OR	*P*-value^a^	Adjusted OR	*P*-value^b^
	(*n* = 43)	(*n* = 44)	(95% CI)^a^		(95% CI)^b^	
Any stage	23 (53.5)	12 (27.3)	0.326 (0.130–0.785)	**0.014**	0.196 (0.038–0.843)	**0.036**
Stage 1	11 (25.6)	8 (18.2)				
Stage 2	8 (18.2)	3 (6.8)				
Stage 3	4 (9.3)	1 (2.3)				

ACP, antegrade cerebral perfusion; RIVP, retrograde inferior vena cava perfusion; OR, odds ratio; CI, confidence interval. The bold values indicates *P* < 0.05.

^a^ Univariate analysis.

^b^ Multivariate analysis adjusted by sex, age, body mass index, hypertension, diabetes mellitus, New York Heart Association class, baseline creatinine level, renal arteries involved in dissection, bypass duration, red blood cell transfusion, lowest nasopharyngeal and rectal temperature.

**Figure 2:**
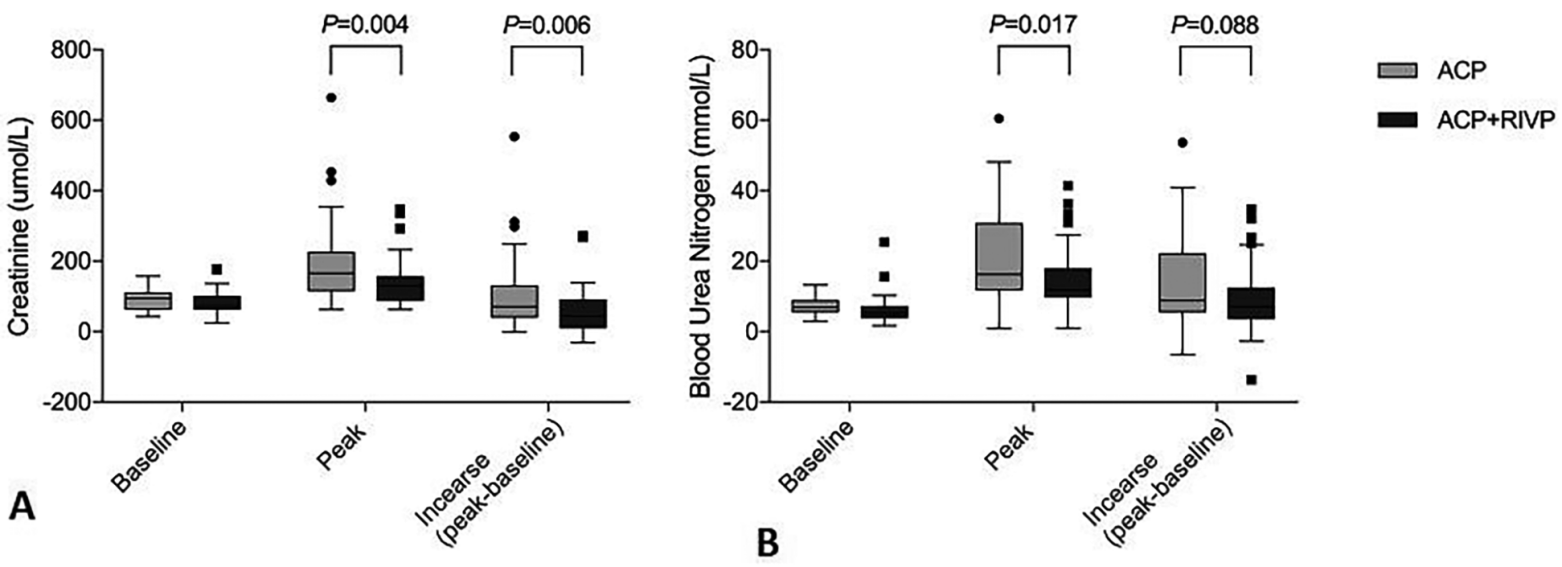
Serum creatinine **(A)** and blood urea nitrogen levels **(B)** in patients undergoing antegrade cerebral perfusion (ACP, *n* = 43) or ACP + retrograde inferior vena caval perfusion (ACP + RIVP, *n* = 44). Shown are the level at baseline, the peak level and the increase (peak—baseline). ACP, antegrade cerebral perfusion; RIVP, retrograde inferior vena caval perfusion.

Patients receiving ACP + RIVP showed a significantly lower postoperative creatinine peak than patients receiving ACP only (139 ± 67 vs. 196 ± 118 *μ*mol/L, *P* = 0.004), as well as smaller serum creatinine increase (58 ± 63 vs. 106 ± 103 μmol/L, *P* = 0.006; Figure 2A). ACP + RIVP patients also showed lower peak blood urea nitrogen (15 ± 9 vs. 21 ± 13 mmol/L, *P* = 0.017; Figure 2B).

Other outcomes occurred in 4 out of 87 (4.6%) of whom experienced all-cause death; 8 (9.2%), stroke; 51 (58.6%), prolonged ventilation. ACP + RIVP was associated with lower risk of prolonged ventilation than ACP only (crude OR 0.361, 95% CI 0.146–0.859, *P* = 0.023), but this risk reduction remained insignificant (adjusted OR 1.231, 95% CI 0.319–5.067, *P* = 0.765) after adjusting (Supplementary Table S4).

### RIVP for metabolism during circulatory arrest and AKI

**Figure 3:**
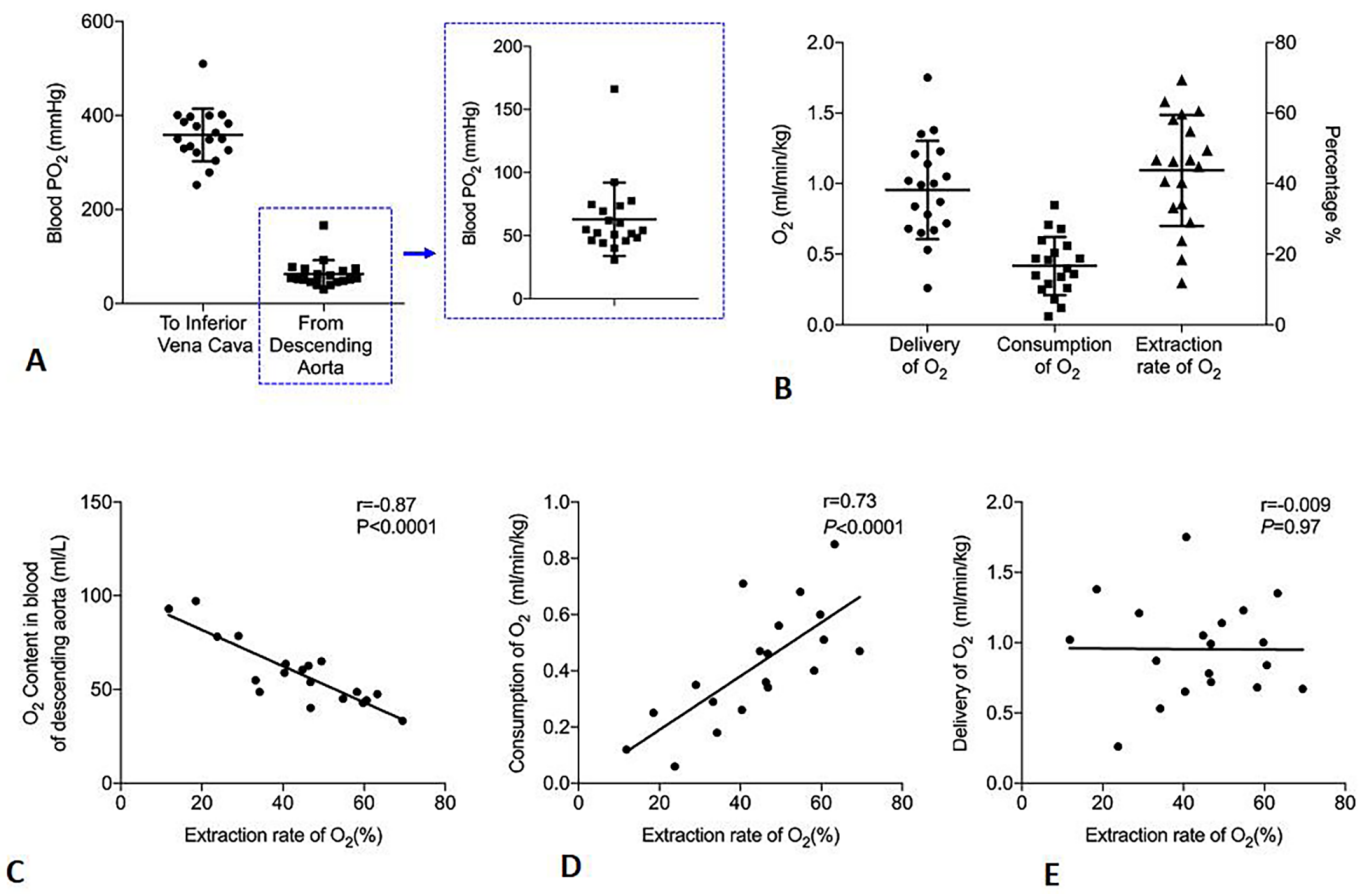
Oxygen (O_2_) metabolism in patients undergoing antegrade cerebral perfusion and retrograde inferior vena caval perfusion (ACP + RIVP) during circulatory arrest (*n* = 19). **(A)** Oxygen partial pressure (PO_2_) in perfused blood of inferior vena cava and returned blood from descending aorta. Data points for the descending aorta are shown in greater detail in the zoomed-in view on the right. **(B)** Oxygen delivery (DO_2_), oxygen consumption (VO_2_), and oxygen extraction ratio (ERO_2_). The *y*-axis on the right indicates the percentage of oxygen extraction ratio (ERO_2_). **(C–E)** Correlations of indices with oxygen extraction ratio (ERO_2_).

In 19 patients in whom blood gas analysis was performed, RIVP blood showed a PO_2_ of 359 ± 57 mmHg and an O_2_ content of 107 ± 14 ml/L; these values decreased to 64 ± 30 mmHg (range 30–166 mmHg) and 59 ± 18 ml/L (range 33–93 ml/L) in the blood returning from the descending aorta (Figure 3A). Based on SO_2_, PO_2_ and RIVP flow, we calculated DO_2_ to be 0.98 ± 0.34 ml/min/kg (range 0.26–1.75 ml/min/kg) and VO_2_ to be 0.43 ± 0.2 ml/min/kg (range 0.06–0.85 ml/min/kg). This corresponded to a mean ERO_2_ of 44 ± 16% (range 11.8–69.5%) (Figure 3B). The extraction ratio correlated strongly with residual oxygen in returned blood from the descending aorta (*r* = −0.87, *P* < 0.001) and with VO_2_ (*r* = 0.73, *P* < 0.001) but not with DO_2_ (*r* = −0.009, *P* = 0.97) (Figures 3C–E).

**Figure 4:**
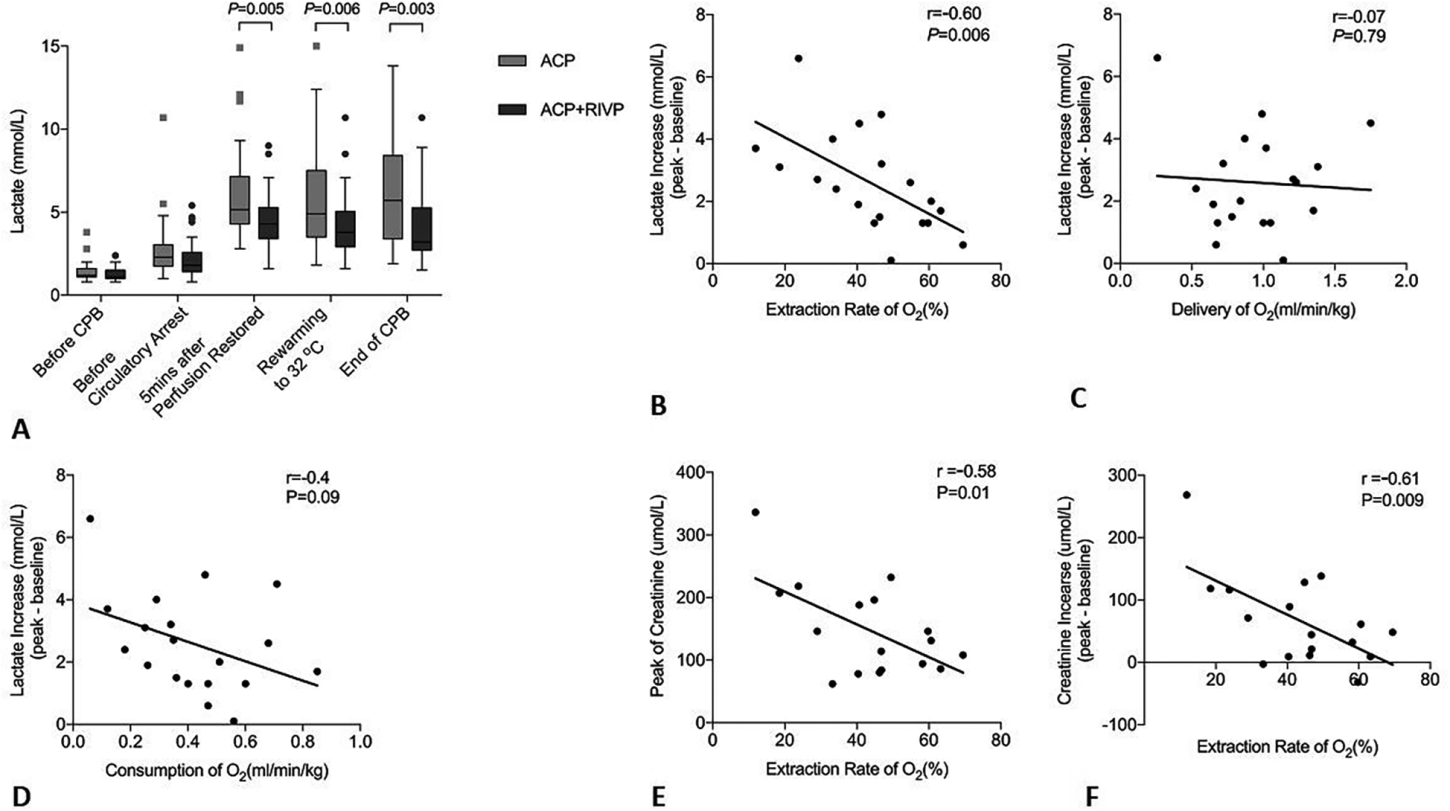
Oxygen metabolism and renal function. **(A)** Lactate levels at different timepoints during surgery in patients who underwent antegrade cerebral perfusion only (ACP, *n* = 43) or ACP + retrograde inferior vena caval perfusion (ACP + RIVP, *n* = 44). **(B–D)** Correlations between lactate increase (peak—baseline) and various oxygen indices in 19 patients who underwent ACP + RIVP. **(E,F)** Negative correlation of oxygen extraction ratio (ERO_2_) with peak creatinine and creatinine increase in 19 patients who underwent ACP + RIVP. In these experiments, lactate peak and increase were defined with respect to the perioperative peak, while creatinine peak and increase were defined with respect to the postoperative peak. CPB, cardiopulmonary bypass; ACP, antegrade cerebral perfusion; RIVP, retrograde inferior vena caval perfusion; O_2_, oxygen.

In the entire study cohort, lactate levels significantly increased after restoration of lower body perfusion, and hyperlacticemia persisted through the end of surgery. The ACP group had higher lactate levels than the ACP + RIVP group starting from 5 min after restoration of lower body perfusion until the end of CPB (Figure 4A).

In RIVP patients, lactate levels were elevated to 4.06 ± 1.68 mmol/L (range 1.6–8.3) at the end of CPB, corresponding to a lactate increase of 2.59 ± 1.61 mmol/L. This increase correlated negatively with ERO_2_ (*r* = −0.6, *P* = 0.006; Figure 4B), but not with DO_2_ (*r* = −0.07, *P* = 0.79) or VO_2_ (*r* = −0.4, *P* = 0.09) (Figures 4C-D).

Potential correlation between metabolic indicators and AKI was explored in blood gas analyses results obtained from the descending aorta during circulatory arrest in ACP + RIVP patients. ERO_2_ during RIVP negatively correlated with the creatinine peak (*r* = −0.58, *P* = 0.01; Figure 4E) and with the increase in creatinine (*r* = −0.61, *P* = 0.009; Figure 4F). In contrast, lactate increase during RIVP showed a weak correlation with creatinine peak (*r* = 0.46, *P* = 0.06) and creatinine increase (*r* = 0.43, *P* = 0.08).

## Discussion

The main finding of this study was that RIVP during circulatory arrest in patients with TARS decreased the risk of AKI, and this decrease was dependent on tissue ERO_2_. Furthermore, we showed that RIVP under 22.5 ± 3.8 mmHg provided a blood flow of 9.6 ± 3.2 ml/kg/min with a rectal temperature of 27.7 ± 1.4°C. During RIVP under these conditions, PO_2_ in ascending aortic blood was higher than 30 mmHg, suggesting that RIVP can deliver sufficient oxygen to meet the metabolic requirements of vital organs in the lower body.

AAAD patients undergoing CPB for TARS present a high risk of postoperative AKI (4, 8), which likely stems from vascular dissection involving the renal arteries, as well as from ischemic injury during circulatory arrest to allow anastomosis of the graft and descending aorta. To reduce the ischemia of the lower body, various techniques have been applied, including left subclavian artery perfusion (21), balloon occlusion catheter perfusion in the descending thoracic aorta, femoral artery perfusion with antegrade aortic balloon occlusion (22), or intermittent lower body perfusion (23). These methods may be less appropriate for AAAD patients because of the risk of intimal injury and malperfusion (24). In the present study, ischemic injury was attenuated by RIVP, a novel technique without risk of intimal injury and malperfusion that provides blood flow from veins to vital organs in the lower body during circulatory arrest. Combining RIVP with ACP led to significantly lower risk of AKI in this study than ACP alone. Compared with our results published in 2021 (25), however, incidences of AKI in both groups were much reduced.

Similar to retrograde cerebral perfusion, RIVP may exert its protective effects by allowing better cooling and less embolization (26). Because RIVP also provides oxygenated blood to the lower body, we collected blood gas analyses results from RIVP blood and the returned blood from descending aorta in order to calculate DO_2_, VO_2_, and ERO_2_. O_2_ content in the blood that had returned from the descending aorta was much lower than that in RIVP blood. This difference was used to calculate the ERO_2_, which negatively correlated with peaks of lactate and creatinine as well as with increases in creatinine. These results suggest that the protective effect of RIVP on the kidney depends on the ERO_2_ by tissues.

Under normal conditions, VO_2_ remains fairly constant despite changes in DO_2_. If DO_2_ falls below a critical threshold, VO_2_ diminishes as well (15, 27, 28), and anaerobic metabolism occurs. In our study, DO_2_ correlated poorly with VO_2_, ERO_2_, and increases in lactate and creatinine. These observations suggest that, in our patients, lower body metabolism was independent of DO_2_, implying that RIVP delivered adequate amounts of oxygen to the kidney. Consistent with this idea, blood from the descending aorta was still able to release oxygen (PO_2_ > 30 mmHg).

In view that the ERO_2_ widely varied from 12%–69% in our patients, and that AKI was still higher in ACP + RIVP patients than in regular bypass patients, we hypothesize that risk of AKI depends on additional factors besides ischemic injury during circulatory arrest. For example, low ERO_2_ may be due to a decrease in oxygen uptake by tissue, rather than a decrease in DO_2_. Given that low temperature can lead to low ERO_2_ (29), it may be preferable to keep body temperature higher. Future studies should investigate whether RIVP can meet metabolic oxygen demand at higher body temperatures.

Anaerobic glycolysis still occurred during RIVP in our patients, as reflected in the significantly greater lactate levels in blood from the descending aorta than in RIVP blood flow. The fact that lactate levels correlated poorly with creatinine levels after surgery implies that anaerobic metabolism may occur not in kidney tissue but in other tissues such as the legs, which are not reached by RIVP blood flow because of venous valves (30).

This study presents several limitations. First, only 43% of the patients in the ACP + RIVP group were detected the blood gas with samples from the descending aorta for oxygen metabolism analysis, which may have introduced potential bias. Second, we were able to collect information from imaging studies on whether the dissection involved the renal arteries, but did not have information about whether this involvement resulted in malperfusion. Additionally, the volume of contrast agent administered during preoperative imaging studies could not be quantified. Third, the absence of the lowest hemoglobin may limit our ability to fully understand the differences in transfusion rates between the groups and their potential impact on patient outcomes. Fourth, in this study we did not evaluate other complications related to abdominal ischemia, such as paraplegia, gastrointestinal complications and acute liver injury. The reason was that the relatively low incidence of these complications would require a larger sample size to analyze differences between two groups. Finally, although the majority of perioperative variables were comparable between the two groups, minority variables such as coeliac trunk dissection rate, DO_2_ in the post-circulatory arrest period and other unmeasured confounding factors may still affect this retrospective study. Further prospective studies are needed to verify these aspects in the future.

## Conclusion

Despite these limitations, the results of this retrospective study suggest that RIVP can significantly reduce risk of AKI by providing adequate oxygen supply to the lower body during circulatory arrest in patients undergoing TARS.

## Data Availability

The original contributions presented in the study are included in the article/Supplementary Material, further inquiries can be directed to the corresponding authors.
