# Supplementary material for: Retrograde inferior vena cava perfusion reduces the risk of acute kidney injury depending on the oxygen extraction ratio. A retrospective cohort study
Source: Front Cardiovasc Med. 2025 Apr 28;12:1514247. doi: 10.3389/fcvm.2025.1514247 (PMC12066508; doi:10.3389/fcvm.2025.1514247)
Supplement: Supplementary file 1 [file Table1.docx]

**Table S1. Frequency of arterial cannulation techniques used during surgery.**

| **Cannulation** | **ACP**  **(n=43)** | **ACP+RIVP**  **(n=44)** | **P-value** |
| --- | --- | --- | --- |
| Femoral artery | 34 (79.1) | 39 (88.6) | 0.225^*^ |
| Aortic arch | 8 (18.6) | 5 (11.4) | 0.344^*^ |
| Right axillary artery | 1 (2.3) | 0 (0.0) | 0.494^§^ |

Values are shown as n (%).

^*^ P-value from chi-squared test.

^§^ P-value from Fisher’s exact test.

Abbreviations: ACP, antegrade cerebral perfusion; RIVP, retrograde inferior vena cava perfusion.
