# Supplementary material for: Retrograde inferior vena cava perfusion reduces the risk of acute kidney injury depending on the oxygen extraction ratio. A retrospective cohort study
Source: Front Cardiovasc Med. 2025 Apr 28;12:1514247. doi: 10.3389/fcvm.2025.1514247 (PMC12066508; doi:10.3389/fcvm.2025.1514247)
Supplement: Supplementary file 2 [file Table2.docx]

**Table S2. Demographic and clinical characteristics of 87 patients, stratified by perfusion strategy during circulatory arrest.**

| **Characteristic** | **ACP**  **(n=43)** | **ACP+RIVP**  **(n=44)** | **P-value** |
| --- | --- | --- | --- |
| Male | 35 (81.4) | 40 (90.9) | 0.198^*^ |
| Age, years | 44±10 | 46±9 | 0.347 |
| Han Chinese | 40 (93.0) | 42 (95.5) | 0.676^§^ |
| Weight, kg | 72 (64, 80) | 70.3 (65, 82) | 0.958 |
| Body mass index, kg/m^2^ | 25±4 | 25±4 | 0.575 |
| Current smoker | 27 (62.8) | 25 (56.8) | 0.570^*^ |
| Current alcohol consumption | 9 (20.9) | 11 (25.0) | 0.652^*^ |
| **Medical history** |  |  |  |
| Hypertension | 27 (62.8) | 26 (59.1) | 0.724^*^ |
| Diabetes mellitus | 1 (2.3) | 2 (4.5) | 0.999^§^ |
| Hyperlipidemia | 1 (2.3) | 0 (0.0) | 0.494^§^ |
| Pulmonary infection | 2 (4.7) | 1 (2.3) | 0.660^§^ |
| Coronary heart disease | 1 (2.3) | 0 | 0.494^§^ |
| New York Heart Association class, I/II/III/IV | 1/37/3/2 | 0/38/4/2 | 0.766^*^ |
| Left ventricular ejection fraction, % | 60±9 | 60±8 | 0.849 |
| **Preoperative laboratory tests** |  |  |  |
| Hemoglobin values, g/L | 134.7±17.6 | 137.9±18.3 | 0.416 |
| Platelet counts, 10^9^ **/**L | 166 (108.5, 210) | 150.5 (122, 213) | 0.690 |
| Serum creatinine, μmol/L | 90.2±30.0 | 82.6±30.4 | 0.244 |
| Serum creatinine >110 μmol/L | 10 (23.3) | 6 (13.6) | 0.247^*^ |
| **Medications** |  |  |  |
| β-adrenergic receptor blocker | 31 (72.1) | 31 (70.5) | 0.866^*^ |
| Diuretics | 16 (37.2) | 11 (25.0) | 0.218^*^ |
| Aspirin | 1 (2.3) | 1 (2.3) | 0.999^§^ |
| **Arteries involved in dissection** |  |  |  |
| Coronary | 11 (25.6) | 10 (22.7) | 0.756^*^ |
| Brachiocephalic trunk | 27 (62.8) | 34 (77.3) | 0.140^*^ |
| Left common carotid | 21 (48.8) | 22 (50.0) | 0.914^*^ |
| Left subclavian | 21 (48.8) | 29 (65.9) | 0.107^*^ |
| Coeliac trunk | 9 (20.9) | 18 (40.9) | **0.044^*^** |
| Left renal | 10 (23.3) | 14 (31.8) | 0.372^*^ |
| Right renal | 13 (30.2) | 13 (29.5) | 0.944^*^ |

Values are shown as n (%), mean ± standard deviation or median (25th percentile, 75th percentile).

^*^ P-value from chi-squared test.

^§^ P-value from Fisher’s exact test.

Abbreviations: ACP, antegrade cerebral perfusion; RIVP, retrograde inferior vena cava perfusion.
