# Supplementary material for: Retrograde inferior vena cava perfusion reduces the risk of acute kidney injury depending on the oxygen extraction ratio. A retrospective cohort study
Source: Front Cardiovasc Med. 2025 Apr 28;12:1514247. doi: 10.3389/fcvm.2025.1514247 (PMC12066508; doi:10.3389/fcvm.2025.1514247)
Supplement: Supplementary file 3 [file Table3.docx]

**Table S3. Perioperative data of 87 patients, stratified by** **perfusion strategy during circulatory arrest.**

| **Variable** | **ACP**  **(n=43)** | **ACP+RIVP**  **(n=44)** | **P-value** |
| --- | --- | --- | --- |
| **Surgical procedure** |  |  |  |
| Ascending aorta replacement | 40 (93.0) | 40 (90.9) | 0.999^§^ |
| Elephant trunk technique | 38 (88.4) | 43 (97.7) | 0.110^§^ |
| Aorta arch reconstruction | 42 (97.7) | 44 (100.0) | 0.494^§^ |
| Brachiocephalic trunk artery reconstruction | 39 (90.7) | 42 (95.5) | 0.434^§^ |
| Left common carotid artery reconstruction | 37 (86.0) | 42 (95.5) | 0.157^§^ |
| Left subclavian artery reconstruction | 24 (55.8) | 35 (79.5) | **0.018^*^** |
| Aortic valve replacement | 21 (48.8) | 18 (40.9) | 0.457^*^ |
| Aortic valvuloplasty | 9 (20.9) | 10 (22.7) | 0.839^*^ |
| Coronary artery bypass grafting | 14 (32.6) | 13 (29.5) | 0.761^*^ |
| **Pre-circulatory arrest period** |  |  |  |
| DO_2_, mL/kg/min | 5.6±1.0 | 5.8±0.9 | 0.167 |
| **Post-circulatory arrest period** |  |  |  |
| DO_2_, mL/kg/min | 5.4±1.3 | 6.2±1.0 | **0.001** |
| **Circulatory arrest period** |  |  |  |
| Antegrade cerebral perfusion flow, mL/kg/min | 6.4±2.1 | 7.2±2.2 | 0.112 |
| **Retrograde inferior vena cava perfusion** |  |  |  |
| Flow, mL/kg/min | - | 9.6±3.2 | - |
| Pressure, mmHg | - | 22.5±3.8 | - |
| **Lowest temperature, ^°^C** |  |  |  |
| Nasopharyngeal | 24.7±1.3 | 26.4±1.6 | **<0.001** |
| Rectal | 26.3±1.6 | 27.7±1.4 | **<0.001** |
| **Time, min** |  |  |  |
| Circulatory arrest time | 33.4±9.6 | 35.1±6.9 | 0.334 |
| Cross-clamping time | 183±55 | 183±43 | 0.999 |
| Cardiopulmonary bypass duration | 276±58 | 250±53 | **0.031** |
| **Medications** |  |  |  |
| Tranexamic acid, g | 1 (0, 1) | 1 (0, 1) | 0.762^†^ |
| Prothrombin complex, units | 600 (600, 600) | 600 (600, 600) | 0.103^†^ |
| Fibrinogen, g | 1 (1, 1) | 1 (1, 1) | 0.843^†^ |
| **Blood transfusion, units** |  |  |  |
| Red blood cells | 3 (0, 5) | 0 (0, 3) | **0.010**^†^ |
| Fresh frozen plasma | 1.8 (0, 3.0) | 1.6 (0, 2.9) | 0.947^†^ |
| Platelets | 2 (2, 2) | 2 (2, 2) | **0.032**^†^ |

Values are shown as n (%), mean ± standard deviation or median (25th percentile, 75th percentile).

Abbreviations: ACP, antegrade cerebral perfusion; RIVP, retrograde inferior vena cava perfusion.

^*^ P-value from chi-squared test.

^§^ P-value from Fisher’s exact test.

^†^ P-value from rank sum test.
