# Supplementary material for: Retrograde inferior vena cava perfusion reduces the risk of acute kidney injury depending on the oxygen extraction ratio. A retrospective cohort study
Source: Front Cardiovasc Med. 2025 Apr 28;12:1514247. doi: 10.3389/fcvm.2025.1514247 (PMC12066508; doi:10.3389/fcvm.2025.1514247)
Supplement: Supplementary file 4 [file Table4.docx]

**Table S4. Postoperative** **outcomes in 87 patients stratified by perfusion strategy.**

| Other outcomes, n (%) | ACP  (n=43) | ACP+RIVP  (n=44) | Crude OR  (95% CI) ^a^ | P-value ^a^ | Adjusted OR  (95% CI) ^b^ | P-value ^b^ |
| --- | --- | --- | --- | --- | --- | --- |
| All-cause death | 3 (7.0) | 1 (2.3) | 0.310  (0.015 to 2.533) | 0.319 | 0.057  (0.001 - 2.296) | 0.175 |
| Stroke | 4 (9.3) | 4 (9.1) | 0.975  (0.217 - 4.385) | 0.973 | 1.563  (0.201 - 11.69) | 0.658 |
| Prolonged ventilation | 30 (69.8) | 21 (47.7) | 0.361  (0.146 - 0.859) | **0.023** | 1.231  (0.319 - 5.067) | 0.765 |

^a^ Univariate analysis.

^b^ Multivariate analysis adjusted by sex, age, body mass index, hypertension, diabetes mellitus, New York Heart Association class, baseline creatinine level, renal arteries involved in dissection, bypass duration, red blood cell transfusion, lowest nasopharyngeal and rectal temperature.

Abbreviations: ACP, antegrade cerebral perfusion; RIVP, retrograde inferior vena cava perfusion; OR, odds ratio; CI, confidence interval.
